# Jens Lunde and Christine Whitehead: Milestones in European housing finance: John Wiley & Sons, West Sussex, United Kingdom, 2015, 488 pp, ISBN: 978-1-118-92945-2

**DOI:** 10.1007/s10901-017-9577-y

**Published:** 2017-11-15

**Authors:** Susan M. Wachter

**Affiliations:** The Wharton School, University of Pennsylvania, Philadelphia, PA USA

While the global focus has been on the U.S. subprime crisis and after effects, the housing finance systems of Europe have also experienced radical changes in recent decades. This book, edited by Jens Lunde and Christine Whitehead, is a must-read for those seeking to understand the milestones in European housing finance (as it is titled).

The volume was initiated on the occasion of the 25th anniversary of the European Network on Housing Research (ENHR)’s Working Group on Housing Finance. Hence the involvement of housing finance specialists from 21 European countries (and Australia) who are experts in their country’s housing markets and institutions. A major strength of the volume is their knowledge of housing finance institutions and political, social and economic contexts of their particular countries. Country specialists will find this book to be a go-to resource not only for understanding their country’s housing finance and economic markets history but also for insights into why these markets evolved in the manner they did, their likely future trajectory and policy issues that need to be addressed.

A second important contribution of the book is the survey deployed to compare institutions and outcomes in the years 1989 and 2014; the survey provides information on the detailed features of each country’s mortgage system in 1989 and again in 2014. Authors are also asked to provide the milestones in the evolution of their country’s housing finance system. The result is a comprehensive view of the rapid changes that have taken place in the last quarter century across Europe.

The period covered is serendipitous since the last quarter century has proved to be a period of great change which calls out for in-depth analysis such as is provided here. The period includes a high degree of financial innovation, amid an increasingly significant level of European and global economic interconnectedness and several major economic cycles, including the recent crisis. In particular the focus on these years is critical for understanding the path from the regulated “special circuit” systems that prevailed prior to the 1980s to the deregulated housing finance systems of the 1990s and early 2000s. Financial liberalization and declining interest rates caused the volume of mortgage finance to double in the first 10 years of this period and then double again in the next decade, with the overall ratio of mortgage debt to GDP, along with housing prices (relative to income) increasing dramatically in the aggregate. Nonetheless the evolution of housing finance is by no means a story of the triumph of deregulation, as this period includes the 2007–2009 Global Financial Crisis (GFC), the worst crisis since the Great Depression. Nor is it a single story. Several European countries, specifically, Ireland, Spain and Iceland, had housing booms and busts and subsequent GDP declines in the recession of 2009–10 larger than the US GDP decline. Others, for example, Germany and France were mostly unscathed. Why is this?

The consistent, methodological approach employed in each chapter helps to inform comparative study of these vastly different outcomes over this period. Much can be learned by these comparisons, most importantly for analyzing the impact of factors that extend beyond geography—including market liberalization, the role of government and the historical evolution of political systems—dynamics that are particularly important in the context of a topic as broad as housing finance. For example, within the English-speaking countries included in this volume (Australia, Ireland and the United Kingdom) are the economies with the most market-oriented housing finance systems. They are also similar in the structure of their financial systems, particularly their reliance on retail deposit funded bank lending and short term variable rate mortgages. Despite these common features, Australia experienced a boom with (so far) no bust while the UK and especially Ireland experienced housing booms and busts. On the other hand, in Germany and Austria, countries with ‘corporatist’ housing finance systems, experienced no rise in housing prices.

This is also the period of transformation in formerly socialist countries. The chapters on countries that were formerly defined by socialist systems (the Czech Republic, Poland, Hungary, Russia and Slovenia) provide a view of the development of housing finance systems in newly emergent market systems. The ‘turn to markets’ in many Eastern European countries after the decline of the Soviet Union has been one of the most significant global economic events during the period in focus. But once again important differences occur across countries. Several (Poland; Hungary; Slovenia) took advantage of foreign exchange-denominated mortgages, during the GFC, suffered accordingly with exchange rate devaluation. With housing finance markets undeveloped, Russia remained relatively unscathed by the financial turmoil.

The first two chapters of Lunde and Whitehead’s volume—written by the editors themselves—provide both an overview of the book’s purpose as well as an understanding of the period in focus (1989–2014). By establishing a framework for understanding the trends across European housing finance systems, the editors provide a basis for comparative analysis between countries. The volume’s penultimate chapter, co-authored by Jennifer Johnson, Lorenzo Isgrò and Sylvain Bouyon, provides a useful descriptive overview of the changes that have affected the European continent—particularly the formation of the EU—and their implications for housing finance and the current standing of regulatory change.

The volume’s concluding chapter, authored by Lunde and Whithead, adopts a long view perspective that focuses on both historical developments in housing finance as well as the outlook for the future of these systems. Lunde and Whitehead address several key themes that characterize this quarter century. Specifically, they observe that, for almost all the countries included in the volume, mortgage markets have deepened—meaning that overall mortgage debt has increased substantially. This development can be viewed as positive in terms of access to housing finance, and thus, housing opportunity. Nonetheless, Lunde and Whitehead acknowledge the role of mortgage debt in the GFC and conclude with marked uncertainty about the future of housing finance across Europe.

Two broad policy issues about the future trajectory of housing are posed. First, the increased access to mortgage finance has been accompanied and has contributed to the historic rise in house prices and limited access to housing for many. Second, the project to protect economies from systemic risk arising from housing finance is far from complete. Bank regulators and policy makers in Europe are evaluating different approaches to mortgage markets, including secondary markets. Central banks are implementing various approaches to macro prudential policy throughout the continent. Nonetheless, the vulnerability of the banking system to house price bubbles and to macro shocks, for example, through an unexpected rise in interest rates, remains.

The publication of this volume coincides with a period of implementation and design of the reregulation of housing finance. US policy makers are calling into question the necessity of some of the heightened regulation under the Dodd Frank Act. European nations must still implement finance regulation, despite the broad agreements under Basel III. The realm of housing finance regulation affects a nation’s economy and the well-being of its population. As such, Lunde and Whitehead’s contribution will be important as the debate on the future of the structure of housing finance systems continues.

